# Development and Characterization of an Enzyme Membrane Reactor for Fructo-Oligosaccharide Production

**DOI:** 10.3390/membranes9110148

**Published:** 2019-11-10

**Authors:** Jan Philipp Burghardt, Luca Antonio Coletta, Ramona van der Bolt, Mehrdad Ebrahimi, Doreen Gerlach, Peter Czermak

**Affiliations:** 1 Institute of Bioprocess Engineering and Pharmaceutical Technology, University of Applied Sciences Mittelhessen, 35390 Giessen, Germany; 2 Faculty of Biology and Chemistry, Justus-Liebig University of Giessen, 35390 Giessen, Germany; 3 Fraunhofer Institute for Molecular Biology and Applied Ecology (IME), Project Group Bioresources, Heinrich-Buff-Ring 26, 35392 Giessen, Germany

**Keywords:** ceramic membrane, enzyme membrane reactor (EMR), fructo-oligosaccharides (FOS), prebiotic nutrition, *Kluyveromyces lactis*, resistance-in-series model, flux modeling, complete pore blocking

## Abstract

Fructo-oligosaccharides (FOS) are linear fructans comprising 2–5 fructose units linked to a terminal glucose residue. They are widely used as food and feed additives due to their sweetness, low calorific value, and prebiotic properties. Here we describe the synthesis of FOS catalyzed by a cell-free crude enzyme solution containing recombinant fructosyltransferase (1-FFT) produced in the yeast *Kluyveromyces lactis*. During the enzyme catalysis, glucose accumulates as a by-product and eventually inhibits FOS production. We therefore used an enzyme membrane reactor (EMR) to achieve the continuous removal of glucose and the simultaneous replenishment of sucrose. We observed a loss of flux during the reaction with the characteristics of complete pore blocking, probably caused by a combination of proteins (enzyme molecules) and polysaccharides (FOS). Such complex fouling mechanisms must be overcome to achieve the efficient production of FOS using EMR systems.

## 1. Introduction

Fructo-oligosaccharides (FOS) are linear fructans containing 2–5 β(2→1)-linked D-fructose units joined to a terminal α(1→2)-linked glucose residue. FOS are water-soluble molecules that have a low calorific value but 30–50% of the sweetness of sucrose, depending on their chemical structure and degree of polymerization [1,2]. Short-chain FOS in particular have similar organoleptic properties to sucrose and are widely used as low-calorie sweeteners [3]. FOS also have prebiotic properties, stimulating the proliferation of bifidobacteria in the large intestine and thus supporting the immune system [4,5]. FOS occur naturally in many plants, including onions, bananas, artichokes, tomatoes, and asparagus. They can be extracted from these sources or produced by the limited enzymatic hydrolysis of inulin, which is abundant in chicory and Jerusalem artichoke [6,7]. FOS can also be produced de novo by the enzymatic biotransformation of sucrose. Two classes of enzymes are suitable for the industrial production of FOS: β-fructofuranosidases (EC 3.2.1.26), also known as invertases, and fructosyltransferases (1-FFT) (EC 2.4.1.9). The transferase activity of 1-FFT, which has been isolated from *Aspergillus niger, Aspergillus terreus,* and *Aureobasidium pullulans,* among other sources, is stronger than that of β-fructofuranosidases [8,9,10,11]. The enzymatic synthesis of FOS mainly produces a mixture of 1-kestose (GF_2_), nystose (GF_3_), and 1F-fructofranosylnystose (GF_4_).

One of the drawbacks of enzymatic synthesis is the high cost of enzymes and the loss of soluble enzymes during the reaction. This can be addressed by enzyme immobilization, which allows the extended use of enzymes to maximize their activity. Enzymes can also be retained by porous ceramic membranes, giving rise to enzyme membrane reactors (EMRs) that allow the retention and reuse of the enzyme once the reaction products have been removed. Continuous EMR techniques generally use ultrafiltration (UF) membranes to separate the enzyme from the product stream so that the enzyme can be reused [12].

Any EMR system must address the challenge of maintaining a high permeate flow. Flux generally declines during UF due to a combination of concentration polarization, cake formation, and irreversible membrane fouling [13,14]. Concentration polarization is a natural consequence of membrane selectivity. Solutes that cannot pass through the pores accumulate and form a mass transfer boundary layer at the membrane surface. The osmotic pressure difference between the filtrate and feed solution reduces the effective transmembrane pressure (TMP) and causes the flux to decline [15]. Furthermore, solute molecules accumulating at the membrane surface also reduce the solvent activity and thus the flow through the membrane [16]. Concentration polarization is unavoidable, but it can be abolished by removing the accumulated solute [17]. In contrast to concentration polarization, cake formation involves the accumulation of suspended particles to form a deposit called a filter cake, which acts as an additional filter barrier. Cake formation is also known as reversible fouling because the effects can be abolished by mechanical rinsing and cleaning [18]. Irreversible fouling involves the accumulation of adsorbed molecules and particles on the membrane surface that cannot be removed by rinsing or cleaning, and this causes a permanent decline in flux [19]. Fouling inhibits the flux through porous membranes by various mechanisms, including irreversible or reversible steric pore blocking, pore narrowing by adsorption within the pore structure, and filter cake formation [20,21,22,23].

Some substances and classes of substances are known foulants [24], and if the presence of these substances is unavoidable then certain steps can be taken to reduce or eliminate their effects, such as membrane selection, feed pre-treatment, and the adoption of particular cleaning strategies and/or operating parameters. The mechanisms that cause fouling are often evaluated using the resistance-in-series model [25], which states that the permeate flow decreases due to the increase in fouling-induced resistance. Several different forms of hydraulic resistance can arise during filtration, depending on the type of fouling [26].

When testing a ceramic EMR for the production of FOS, preliminary experiments revealed a rapid decline in flux that made the process unprofitable for long-term continuous operation [1]. In this study, we therefore set out to determine optimal process parameters that improve flux and enzyme activity, thus reducing the residence time of the substrate solution in the EMR system. The target residence time is 1–2 h because most substrate conversion is complete after 2 h, reflecting the inhibitory effect of glucose that accumulates during the reaction [8]. We therefore determined the optimal TMP, followed by a design of experiments (DoE) approach (D-optimal design) to investigate dependency relationships between the permeate flux and enzyme activity on one hand and the TMP and cross-flow velocity (CFV) on the other. We then attempted to identify and exclude fouling mechanisms responsible for the loss of flux during filtration using the center point and statistically optimized confirmation runs of the D-optimal design as model points. Finally, we used the results from the statistical experimental designs to establish a continuous FOS production system. The aim was to increase the FOS yield by feeding the EMR with enzyme solution to replace enzyme molecules that are lost during the reaction.

## 2. Theoretical Aspects

Membrane fouling is a complex process influenced by many mechanisms, but a detailed prediction of flux is useful for EMR design. We used the resistance-in-series model, which states that the permeate flow decreases due to the cumulative effects of phenomena such as concentration polarization, gel layer formation, adsorption, and cake buildup [25]. The permeate flow is reduced by the combination of these resistances, which can be expressed as the total resistance R_total_ (m^−1^) using Equation (1):

(1) $$R_{\mathrm{total}}\;=\;\frac{\Delta P}{\eta \;\times \;J}$$

where J is the permeate flux (m³ m^−2^ s^−1^), η the permeate dynamic viscosity (Pa s), and ΔP is the pressure difference during filtration (Pa). The resistance-in-series model considers pressure in SI units, whereas our experimental results are presented as the TMP in (bar) and the Flux (L m^−2^ h^−1^) across the active membrane surface, to ensure our data are comparable with earlier studies.

The total resistance is typically dependent on the properties of the membrane, such as pore size, porosity, charge and hydrophobicity. Equation (1) can therefore be extended by assigning values to different forms of resistance: membrane resistance (R_m_), and additional resistance due to adsorption (R_a_), concentration polarization (R_cp_), and the gel layer (R_g_), as shown in Equation (2):

(2) $$J\;=\;\frac{\Delta P}{{\eta \;(R}_{m}{+\;R}_{a}{+\;R}_{\mathrm{cp}}{+\;R}_{g})}$$

During filtration, particles settle on the membrane surface to block the pores and/or form a filter cake, both of which increase the resistance. To describe these phenomena in more detail, different models have been developed for dead-end and cross-flow filtration. The mathematical formulation for dead-end filtration [27] describes the decline in flux at constant TMP. The constant n depends on the fouling mechanism, as shown in Equation (3):

(3) $$\frac{d^{2}t}{{\mathrm{dV}}^{2}}\;=\;k{\left(\frac{\mathrm{dt}}{\mathrm{dV}}\right)}^{n}$$

The values for n are 2, 1.5, 1, or 0 for complete pore blocking, standard pore blocking, intermediate pore blocking, and cake filtration, respectively [28]. The corresponding Equations (4)–(7) are summarized in Table 1. J_0_ is defined as the initial flux at t = 0, and k as the fouling-dependent coefficient.

The model for dead-end filtration [27] has been extended by introducing terms for the convective reduction of the fouling layer [29], thus adapting the model for flux decline during cross-flow filtration. The term J_ss_ represents the flux that the system achieves when a steady-state state is reached, as shown in Equation (8):

(8) $$-\frac{\mathrm{dJ}}{\mathrm{dt}}\;\left({\;J}^{n-2}\right)\;=\;k\;\left(J\;-{\;J}_{\mathrm{ss}}\right)$$

Analogous to the dead-end filtration model, Equations (9–12) account for the different fouling mechanisms during cross-flow filtration (Table 2).

## 3. Materials and Methods 

As previously described, we used a *Kluyveromyces lactis* GG799 strain (New England Biolabs, Frankfurt, Germany) for enzyme production [10]. Briefly, the yeast was used to express an *Aspergillus terreus* fructosyltransferase (1-FFT) [9,10].

### 3.1. Enzyme Production

The yeast was cultivated in a Labfors 3 (Infors, Bottmingen, Switzerland) using a previously described FM22 medium [11]. The cultivation and cell-harvest were conducted following previously described conditions [10]. The cell-free supernatant containing the secreted 1-FFT [9,11] was used as a crude enzyme solution. The optimal conditions for 1-FFT activity (pH 5.8 and 70 °C) were reported previously [10].

### 3.2. Filtration Setup

A tubular ceramic membrane was purchased from atech innovations (Gladbeck, Germany) and its properties are summarized in Table 3. An FCPA 80B-4/HE rotary vane pump (AFT, Rosstal, Germany) was used to achieve constant circulation. The filtration medium was heated in a double-walled beaker using an ECO RE 420 thermostat (Lauda, Lauda-Koenigshofen, Germany). 

### 3.3. Estimation of Pure Water Flux

The pure water flux was used to assess the efficiency of cleaning and was measured before and after each filtration. The membrane was flushed with pure water at a CFV of 0.5 m s^−1^. The permeate volume flow was measured at TMPs of 0.5, 1, 1.5, and 2 bar at 25 °C by collecting the filtrate in a graded cylinder.

### 3.4. Cleaning the Column

In order to restore the original filtration performance of the membrane, we tested different cleaning times and different combinations of two cleaning reagents: 1% (*w*/*v*) P3 Ultrasil 14 (Ecolab Deutschland, Monheim am Rhein, Germany) and 1% (*w*/*v*) citric acid (Merck). The different cleaning strategies are shown in Table 4. The cleaning strategy which achieved the highest flow rate was then applied after each filtration. Before and after the application of each cleaning reagent, the membrane was rinsed with deionized water. The purification steps were always performed at 50 °C, 0.75 bar TMP, and a CFV of 0.7 m s^−1^.

### 3.5. DoE Model to Identify Optimal Process Parameters

To set up the DoE model, we staked out the design space by the maximum and minimum process parameters (CFV and TMP). A 900-mL substrate solution (660 g L^−1^ sucrose, 55 mM phosphate buffer pH 5.8) was prepared and preheated in the filtration plant to 70 °C. Following the addition of 100 mL enzyme solution, the permeate flux was measured as described above for the pure water flux. The temperature was recorded on both sides of the membrane, and on the membrane housing. On the permeate side, samples were taken for UHPLC analysis to determine the quantity of FOS formed during the enzyme reaction. In the second step, we characterized the properties of the filtration process using response surface methodology with a D-optimal design. We selected a quadric and randomized design with 18 runs, including four center points. The TMP and CVF were used as parameters to predict the permeate flux after filtration for 8 h and the enzyme activity during the filtration. We selected the TMP range 0.5–5.0 bar and the CFV range 0.5–0.86 m s^−1^. Triplicate confirmation runs were used to confirm the predicted model and the resulting optimized process parameters.

### 3.6. Continuous FOS Production in the EMR

**Figure 1:**
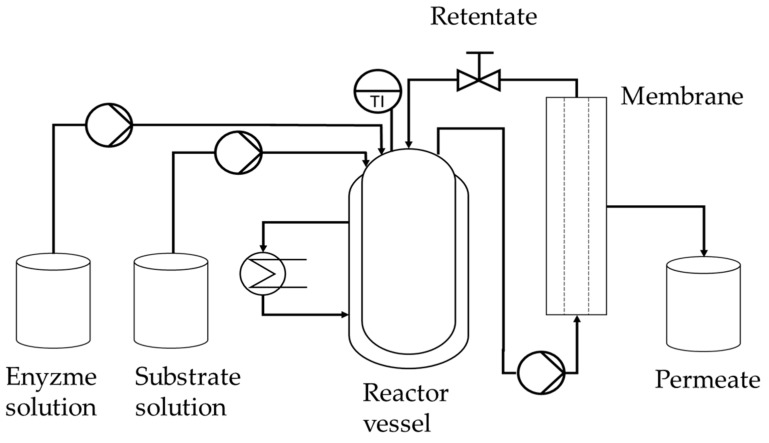
Process scheme for continuous fructo-oligosaccharide (FOS) production containing the enzyme and substrate feed tanks, the reactor vessel, the membrane module, and the permeate tank.

Having confirmed the optimal process parameters in total-recycle mode, we then started the production of FOS in continuous mode (Figure 1). At the beginning of each continuous catalytic reaction, the membrane was preheated as described above. Catalysis was then initiated in total-recycle mode by setting the initial parameters defined in the DoE confirmation run. After 1 h, the system was switched to continuous operation. We fed the EMR with 600 g L^−1^ sucrose in 50 mM phosphate buffer (pH 5.8) using a peristaltic pump (Watson Marlow, Rommerskirchen, Germany) at a flow rate of 16.7 or 8.3 mL min^−1^ corresponding to residence times of 1 and 2 h, respectively. 

The enzyme feed was adjusted using an IPC peristaltic pump (Ismatec, Wertheim, Germany) controlled with Labvision software (HiTec Zang, Herzogenrath, Germany) according to the loss of enzyme during filtration.

### 3.7. Enzyme Stability Analysis 

We also carried out filtration experiments with pure enzyme solution in order to investigate enzyme stability and fouling based on the shear stress generated at different TMP and CFV settings. The reference was a filtration experiment with a CFV of 0.5 m s^−1^ and no external TMP. Two further experiments were performed, the first at CFV = 0.5 m s^−1^ and TMP = 4 bar, and the second at the optimized settings in the DoE conformation run (CFV = 0.68 m s^−1^ and TMP = 0.5 bar). Permeate and retentate samples were taken hourly for SDS-PAGE analysis and bicinchoninic acid (BCA) assays to determine the protein concentration. The enzyme activity was also determined by incubating 100 µL of the enzyme solution from the filtration setup with 900 µL of the substrate for 2 h at 70 °C. 

### 3.8. SDS-PAGE

For SDS-PAGE, 14-µL samples mixed with 5.3 µL Laemmli buffer and 0.6 µL 2-mercaptoethanol were heated to 95 °C for 5 min. After cooling, 12-µL aliquots were transferred to the wells of TGX (Tris-Glycine eXtended) 4–20% gels (Bio-Rad Laboratories, Hercules, CA, USA) in an electrophoresis chamber filled with 1:10 diluted TGS buffer (Bio-Rad Laboratories, Hercules, CA, USA). Quantitative albumin standards of 500, 250, 125, 100, and 50 µg mL^−1^ (Thermo Fisher Scientific, Dreieich, Germany) were run alongside. The gels were analyzed using the ChemiDoc MP imaging system and Image Lab software (Bio-Rad Laboratories, Hercules, CA, USA). To determine the molecular weights of each band, Precision Plus Protein markers (Bio-Rad Laboratories, Hercules, CA, USA) were run in the first and last lanes.

### 3.9. BCA-Assay

Confirmatory protein quantitation was carried out using the Pierce BCA Protein Assay Kit (Thermo Fisher Scientific, Dreieich, Germany) by measuring the adsorption at 562 nm in a 96-well microplate using a Synergy HT plate reader (BioTek, Winooski, VT, USA) [30].

### 3.10. Adsorption of FOS to the Membrane Material

To investigate the adsorption of FOS to the membrane, we carried out catalysis for 8 h at the 1‑L scale using a double-jacket beaker under the same conditions as described above for total-recycle mode. The reaction was started with 600 g L^−1^ sucrose in 50 mM phosphate puffer (pH 5.8) and a 10% (*v*/*v*) enzyme solution. After 8 h, the entire batch was incubated with 5 g Protran BA 85 nitrocellulose (Whatman, Maidstone, UK) overnight to adsorb all soluble proteins. The liquid phase was then tested in the filtration plant running in total-recycle mode (CFV = 0.6 m s^−1^, TMP = 0.5 bar, 70 °C). We recorded the flux, as well as the concentrations of sucrose and FOS. The plant was preheated with water at 70 °C for 1 h.

### 3.11. Model Fitting for Permeate Flux Analysis

The initial parameter estimation for *k* (Table 1 and Table 2) was performed with Matlab R2018b (The MathWorks, Natick, MA, USA) before the models were fitted to the data points using OriginPro 9.0. (OriginLab, Northampton, MA, USA). The coefficient of determination (R^2^) was calculated for each run in order to compare the suitability of the models.

### 3.12. Determination of the Membrane Molecular Weight Cut-off

The molecular weight cut-off (MWCO) of a membrane is the lowest molecular weight at which 90% of the solute is retained [31]. We prepared 1% polyethylene glycol (PEG) [32,33] solutions with molecular weights of 1, 5, 12, 26, and 44 kDa (Sigma-Aldrich Chemie, Taufkirchen, Germany), and filtered them at a TMP of 0.5 bar and a CFV of 0.6 m s^−1^ at room temperature. After 40 min, permeate and retentate samples were taken and passed through a 0.45-μm nylon syringe filter (Carl Roth) prior to HPLC analysis.

The samples were analyzed on an Agilent 1100 HPLC system equipped with an Agilent 1100 series refractive index detector (Agilent Technologies, Waldbronn, Germany). The PEG analytes were separated on Suprema 1000A size-exclusion columns (PSS Polymer Standards Service, Mainz, Germany) and were eluted using a mixture of water and 0.2% (*w*/*v*) NaN_3_ at a flow rate of 1 mL min^−1^ at 60 °C. A standard PEG Kit (PSS Polymer Standards Service) was used for calibration, with molecular weights corresponding to the filtration MWCO values. The retention of each PEG polymer was calculated as the ratio of the peak areas in the permeate and retentate. The data points were fitted by nonlinear regression in OriginPro 9.0. 

### 3.13. Viscosity and Density Measurement

Viscosity was determined using a Haake RS 300 rheometer (Thermo Fisher Scientific) equipped with a Haake DC30 thermostat (Thermo Fisher Scientific) to control the temperature. We also tested the samples using a portable DMA 35 density meter (Anton Paar Germany, Ostfildern, Germany).

### 3.14. UHPLC Analysis

The FOS composition of the samples was determined by UHPLC using a Dionex UltiMate 3000 system equipped with a Corona Veo RS charged aerosol detector (Thermo Fisher Scientific). The solvent was 70% (*v*/*v*) acetonitrile and 30% (*v*/*v*) ultrapure water containing 0.2% (*v*/*v*) triethylamine. An XBridge Amide 3.5 μm column (Waters, Eschborn, Germany) was used to separate the sugars at a constant flow rate of 1 mL min^−1^. Retention times were determined using a FOS standard kit (see above). HPLC analytical-grade sucrose, glucose, and fructose were obtained from Sigma-Aldrich Chemie.

The standard solution was prepared at five dilutions, which were used to prepare calibration curves for each component, allowing the quantification of each component in the filtration experiments. For UHPLC measurement, samples from the enzyme reaction were diluted 1:50 in a 1:1 (*v*/*v*) mixture of acetonitrile and ultrapure water. The samples were then passed through a 0.45-μm nylon filter. The results were evaluated using Chromeleon software (Thermo Fisher Scientific). One unit of enzyme activity (U min^−1^ mL^−1^) was defined as the amount of fructose (µmol) used for the synthesis of FOS per minute related to the used enzyme solution volume. The yield was defined as the ratio of FOS formed, taking into account the number of attached fructose units (degree of polymerization) and the initial sucrose concentration.

## 4. Results

### 4.1. Influence of TMP on Fouling and the Loss of Flux during Filtration

**Figure 2:**
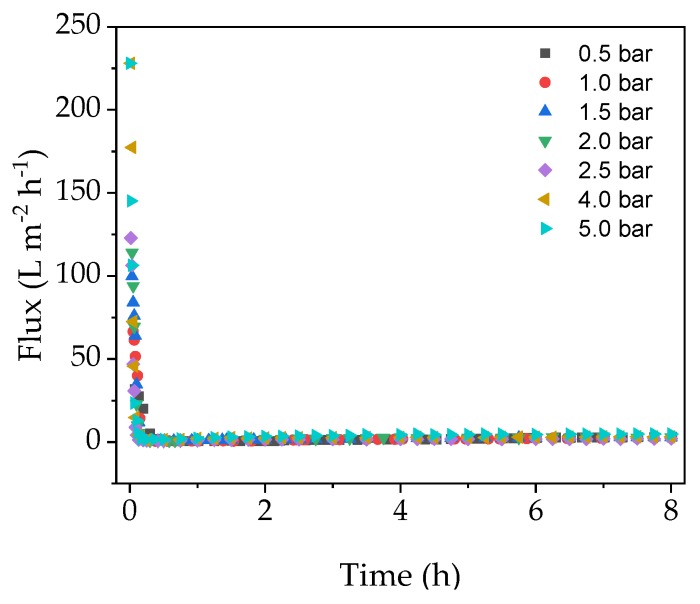
Flux curves for catalysis experiments at different transmembrane pressures (TMPs) (constant 70 °C, cross-flow velocity (CFV) = 0.5 m s^−1^).

We investigated the relationship between TMP, flux and fouling in the EMR in order to determine the conditions associated with the highest final flux after a filtration run lasting 8 h. Figure 2 shows that the flux dropped very sharply during the first 30 min of filtration regardless of the TMP, and remained at a near constant low level until the end of the experiment. 

Although the membrane was heated to 70 °C over a duration of 1 h prior to the experiment, the enzyme solution added at the beginning of the filtration run was stored at 4 °C, leading to a brief drop in temperature. Furthermore, even though the pipes were insulated, heat was nevertheless constantly lost to the environment. The change in membrane temperature caused by these factors may have been responsible for the gradual yet minimal increase in flux after the initial drop (Figure 2). Any temperature-related changes in flux at the beginning of the experiment would be minimal and therefore negligible compared to the effect of fouling. These results suggest that initial flux decline is caused by a strong fouling mechanism, which cannot be avoided by increasing the TMP. Therefore, a critical TMP could not be determined under these conditions.

### 4.2. Optimization of Flux and Enzyme Activity 

**Figure 3:**
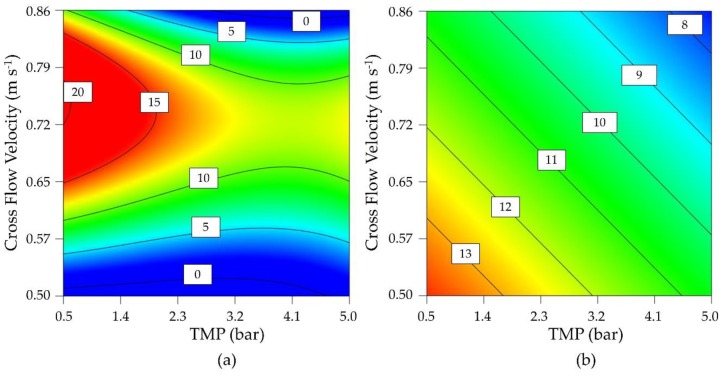
Contour plot of a D-optimal design for the optimization of (**a**) permeate flux (L m^−2^ h^−1^) and (**b**) enzyme activity (U mL^−1^).

Due to the loss of flux under all process conditions during the preliminary tests, parameter optimization was carried out using a DoE approach (D-optimal design) to investigate the influence of TMP and CFV on the permeate flux and enzyme activity. Parameter staking was carried out based on technically achievable process parameters, from which a center point was determined. A moderate CFV showed a positive effect on the flux regardless of the TMP, reflecting its ability to inhibit the formation of a cake layer (Figure 3). However, the flux was reduced regardless of the CFV when the TMP exceeded 3 bar. The greatest possible flux was therefore achieved by lowering the TMP as far as possible while maintaining a moderate CFV.

Enzyme activity was strongly influenced by both TMP and CFV, with low values for both parameters associated with higher maintaining enzyme activity. As a compromise between flux and activity, the operating point at 0.60 m s^−1^ and 0.5 bar was determined using significant cubic and significant linear models for the final flux after 8 h (Figure 3a) and the enzyme activity (Figure 3b). Confirmation runs (*n* = 3) at this process point achieved a flux of 11.87 ± 1.89 L m^−2^ h^−1^ and an enzyme activity of 13.47 ± 0.20 U mL^−1^. The 95% confidence intervals were 7.4–12.5 L m^−2^ h^−1^ and 9.28–16.77 U mL^−1^. As a comparison for later experiments, a flux of 4.24 ± 0.51 L m^−2^ h^−1^ and an enzyme activity of 12.30 ± 0.92 U mL^−1^ were achieved in the center point of the design space (*n* = 4, TMP = 2.75 bar, CFV = 0.68 m s^−1^). We therefore identified two different process points for further analysis: a statistically optimized confirmation run and the center point of the design space. 

### 4.3. Cleaning Strategies

**Figure 4:**
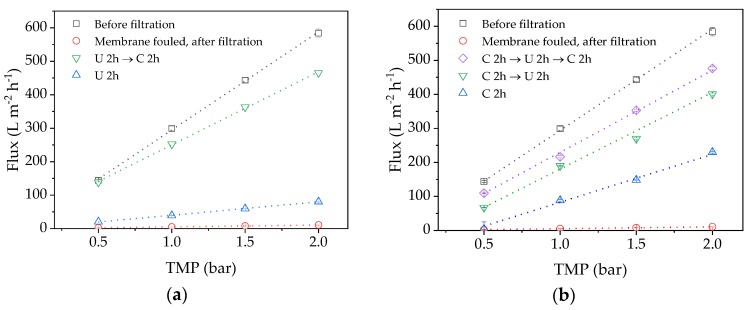
TMP-dependent pure water flux values following different cleaning strategies. (**a**) U 2 h (blue), U 2 h → C 2 h (green); (**b**) C 2 h (blue), C 2 h → U 2 h (green), C 2 h → U 2 h → U 2 h → C 2 h (purple). C = citric acid, U = P3 Ultrasil 14.

Before fouling can be analyzed in detail, the efficiency of cleaning must be investigated to ensure comparable flux conditions. A higher CFV was applied during the cleaning steps because the corresponding higher shear forces can facilitate foulant removal [34]. Figure 4 shows that the pure water flux values declined significantly after the filtration run. After cleaning with either P3 Ultrasil 14 or citric acid for 2 h, the flux recovered only slightly. However, much better results were achieved by applying both cleaning methods in series, particularly if the cleaning step with citric acid for 2 h was repeated (Figure 4b, purple). Because alkaline chemicals remove organic foulants on membranes by hydrolysis and consecutive solubilization, we assume that proteins in the solution can prevent complete membrane penetration by citric acid [34]. These foulants must therefore be removed first, by cleaning with P3 Ultrasil 14, to allow the subsequent application of citric acid to attack additional inorganic foulants such as salt deposits.

Based on these results, if the membrane was first cleaned with P3 Ultrasil 14 and then with citric acid, the cleaning efficiency was 13.8% after the first step and 79.7% after the second. However, if the membrane was cleaned in three steps with citric acid, then P3 Ultrasil 14 and finally with citric acid again, the cleaning efficiencies at each step were 2.8, 46.7, and 76.0%, respectively. Both cleaning procedures therefore achieve a comparable flux, but a sequence of P3 Ultrasil 14 followed by citric acid makes the third cleaning step redundant. It is not possible to completely restore the flux of the membrane indicating there is a degree of irreversible pore blocking.

### 4.4. Calculation of Filtration Resistances according to the Resistance-in-Series Model

Resistance values were calculated using the viscosities and final fluxes in Table 5. The total resistance (R_total_) was determined using the empirical flux values and this included the total fouling resistance as well as the pure membrane resistance. All filtrations were performed at 70 °C, with a CFV of 0.68 m s^−1^ and a TMP of 0.5 bar. The irreversible fouling component was determined by subtracting the pure membrane resistance after one round of cleaning from that of an unused membrane (R_m_). The resistance caused by adsorption and concentration polarization (R_a+cp_) was determined from the flux values of the substrate solution mixed with 10% (*v*/*v*) fresh fermentation medium. The fresh fermentation medium consists only of inorganic salts, vitamins and a carbon source. Therefore the influence of the secreted proteins in the medium on the calculated resistance can be excluded. R_m_ must be subtracted from the obtained value. Because the resulting value is negative, the resistance R_a+cp_ is assumed to be 0. The gel layer resistance (R_g_) can be estimated from the flux values of the 10% (*v*/*v*) enzyme solution. Again, R_m_ must be subtracted to determine the resistance of the gel layer.

**Figure 5:**
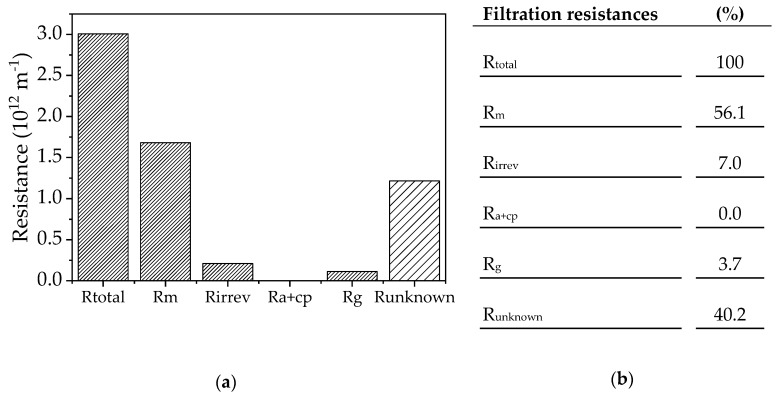
(**a**) Resistance values calculated according to the resistance-in-series model. R_total_ = total resistance; R_m_ = membrane resistance; R_irrev_ = irreversible resistance still present after cleaning; R_a+cp_ = resistance caused by adsorption and concentration polarization of the substrate solution; R_g_ = resistance of the gel layer caused by filtration of the enzyme solution; R_unknown_ = unknown resistance. (**b**) Resistances proportional to the total resistance.

The model solutions were used to calculate the resistances and should reflect the single theoretical resistances as far as possible (Figure 5). This approach showed that the main resistance is only observed when all components of the complete filtration solution are present. The precise division of resistance components is dependent on the viscosities. However, our data indicate that a gel layer formed from the proteins in the filtration solution is not responsible for the observed fouling, and we therefore investigated the source of the fouling phenomenon in more detail.

### 4.5. Maillard Reaction

**Figure 6:**
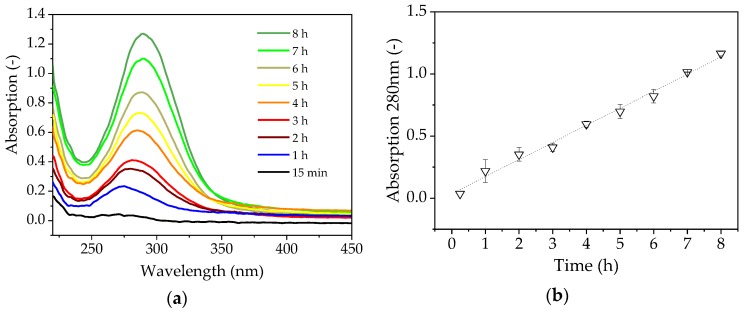
(**a**) Absorption spectrum of enzyme catalysis in the beaker for 8 h. (**b**) Linear increase in absorption at 280 nm.

We observed the browning of the solution during filtration, indicating the formation of by-products via Maillard reactions, which typically occur with reducing sugars at high temperatures [35]. The increase in absorption during the reaction is shown in Figure 6.

The quantity of by-products increases continuously over time, but the rapid flux decrease occurs after just a few minutes and reaches its minimum after 1 h, so the accumulation of brown by-products cannot explain the fouling phenomenon. The spectroscopic measurement of FOS [36], in which the quantity and length of the FOS were not linearly correlated and could not be determined using a single wavelength, shows that the FOS are not responsible for the increase in absorbance (Figure 6). 

### 4.6. Relationship between Time and Flux during the Filtration of a Non-Catalytic FOS Solution

In order to investigate whether FOS adsorb to the membrane during filtration, external enzyme catalysis was carried out in the beaker glass and the reaction solution was filtered over the preheated membrane. Nitrocellulose was included to remove the protein from the solution. The side reactions of the reducing sugars did not allow the protein concentration to be determined using standard detection methods.

**Figure 7:**
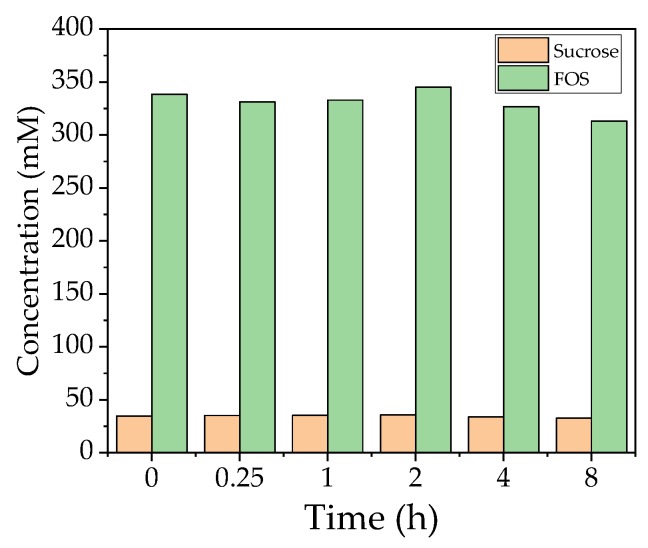
Concentration of total FOS and sucrose in the catalysis reaction during filtration.

The quantity of FOS and sucrose during filtration is shown in Figure 7. The quantity did not change in the first few hours but began to decline towards the end of the run. The sucrose concentration also remained constant. The flux reached 22.67 L m^−2^ h^−1^ after 8 h (70 °C, TMP = 0.5 bar, CFV = 0.6 m s^−1^) and was therefore 191% higher than the confirmation runs under the same conditions. These results indicate that FOS does not adsorb to the membrane and is not responsible for blocking the pores.

### 4.7. Flux Modeling

**Figure 8:**
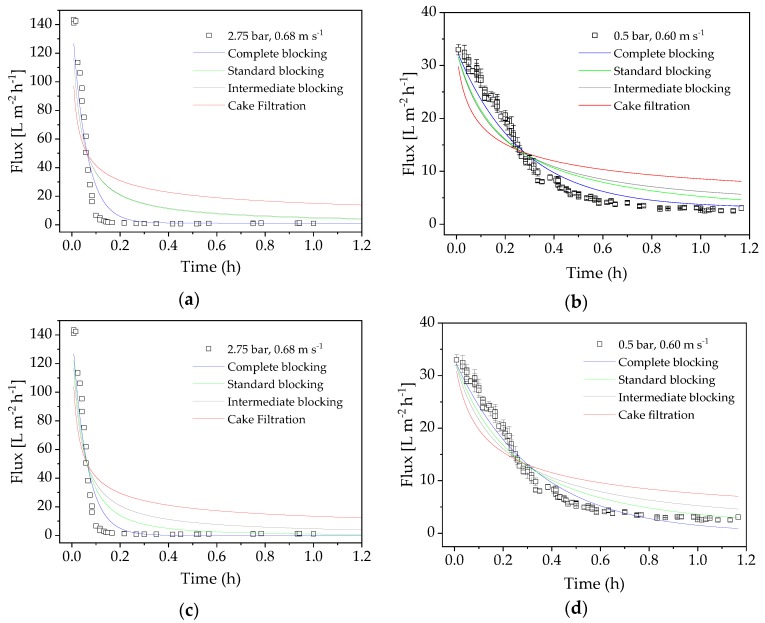
Adaptation of dead-end and cross-flow filtration models. (**a**) Center point (2.75 bar, 0.68 m·s^−1^): dead-end models. (**b**) Optimized confirmation run parameters (0.5 bar, 0.6 m·s^−1^): dead-end models. (**c**) Center point (2.75 bar, 0.68 m·s^−1^): cross-flow models. (**d**) Optimized confirmation run parameters (0.5 bar, 0.6 m·s^−1^): cross-flow models.

Next we compared the flux models of cross-flow and dead-end filtration in experiments carried out under the optimized process conditions of the confirmation run (TMP = 0.5 bar, CFV = 0.60 m s^−1^) and the center point of the DoE (TMP = 2.75 bar, CFV = 0.68 m s^−1^). The model-specific parameter *k* and the coefficient of determination R^2^ were calculated as measures of correlation. Figure 8 shows the flux values of the filtrations for 70 min. The subsequent increase in flux (see Figure 2) reflects the temperature increase caused by heating the filtration equipment, so only the initial section was used for modeling. The course shows the typical UF scenario, with a steep drop in flux followed by a slower decline.

At the beginning of the filtration, high TMP causes the membrane to block almost immediately, resulting in minimal flux. A lower TMP also causes the flux to decrease, even though fouling is delayed (minimal flux at 0.5 bar TMP is reached after ~1 h). The R^2^ of the nonlinear regression of the fouling models (Equations (4)−(7) and (9)–(12)) are listed in Table 6. The values show that the complete blocking model best explains the flux characteristics, and that dead-end filtration models fit best.

Although the cake filtration model has the lowest correlation coefficient, we cannot exclude the possibility that an additional layer of proteins forms on the membrane and increases the resistance. Given that the flux drops more strongly than predicted by the model, we propose that several fouling mechanisms may be acting at the same time.

### 4.8. Enzyme Stability

**Figure 9:**
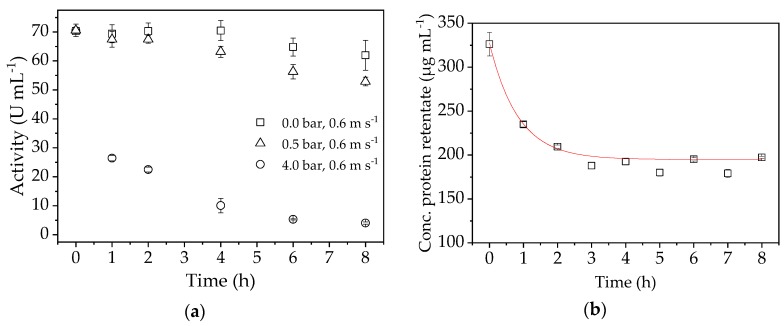
(**a**) Activity of the enzyme during stability tests at TMPs of 4.0, 0.5, and 0 bar. (**b**) Protein concentration in the retentate samples determined by bicinchoninic acid (BCA) assay.

To investigate the stability of the enzyme during filtration in more detail, we carried out further experiments at a CFV of 0.6 m s^−1^ and TMPs of 4, 0.5 and 0 bar. The change in flux over time during these stability tests is shown in Figure 9a. Figure 9b shows the protein concentration course during a filtration under confirmation run conditions (TMP = 0.5 bar, CFV = 0.6 m s^−1^).

The enzyme activity was negatively affected by TMP, and the effect was more severe at 4.0 bar than at 0.5 bar. Without any TMP, the enzyme activity did not decrease by much due to shear forces in the pump and system over the experimental duration, but a clear decrease was observed at 4.0 bar. At 0.5 bar, which is the process parameter optimized for enzyme activity, the loss of activity over time is apparent but less pronounced. This shows that high shear forces in the system triggered by the application of TMP lead to enzyme deactivation. Furthermore, if the enzyme is retained by the membrane, the concentration at the boundary layer increases to a critical level that leads to the deposition of macromolecules from the solution near the membrane to form a gel layer. In this case, a higher TMP can increase the gel thickness, which removes enzymes from the retentate solution [37]. The protein concentration in the retentate samples (Figure 9b) indicates the formation of a gel layer on the membrane. The amount of enzyme slowly decreases until only 42.2% of the initial concentration is present after 8 h. This explains the high drop in flux and the decrease in protein concentration during the first minutes of filtration. A lower TMP is therefore better for the operation even if the flux seems lower, because this prolongs enzyme activity. Non-linear regression was used to determine the exponential function (Y = 194.88 + 132.8×exp(−1.19×t) of the enzyme loss, allowing us to compensate by potentially adding the same amount of enzyme to the system.

**Figure 10:**
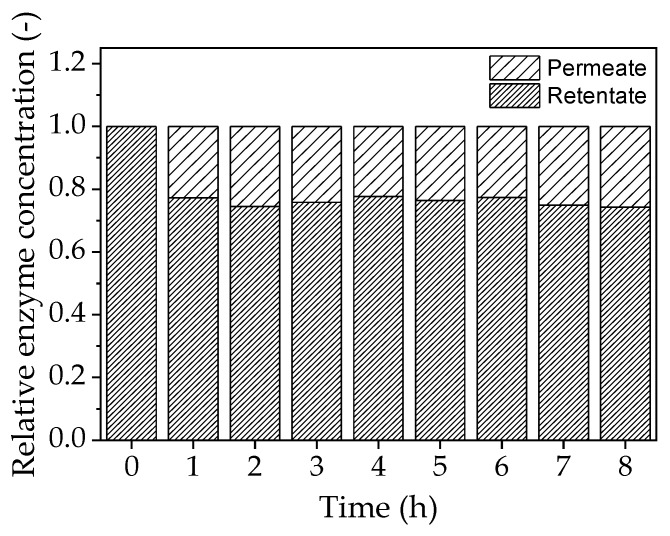
Relative enzyme concentration in the permeate and retentate determined by SDS-PAGE.

Permeate analysis by SDS-PAGE revealed that ~20% of the enzyme crosses the membrane during a filtration run lasting 8 h, even though the molecular mass of the enzyme is larger than the MWCO of the membrane (Figure 10). However, no enzyme activity was detected in the permeate, suggesting that any enzyme molecules therein are completely denatured.

We also conducted a filtration experiment to determine whether the enzyme can form a catalytically active fouling layer. After a filtration under confirmation run conditions the membrane was washed with water for 20 min at 25 °C (TMP = 0.5 bar, CFV = 0.5 m s^−1^).

This was followed by another filtration run lasting 8 h with fresh substrate solution at 70 °C (600 g L^−1^ sucrose, 50 mM phosphate buffer pH 5.8; TMP = 0.5 bar, CFV = 0.6 m s^−1^), but without addition of new enzyme solution. The FOS yield in both the retentate and permeate was <0.05, suggesting that the enzyme does not form a catalytically active fouling layer (data not shown).

### 4.9. Sieve Analysis

**Figure 11:**
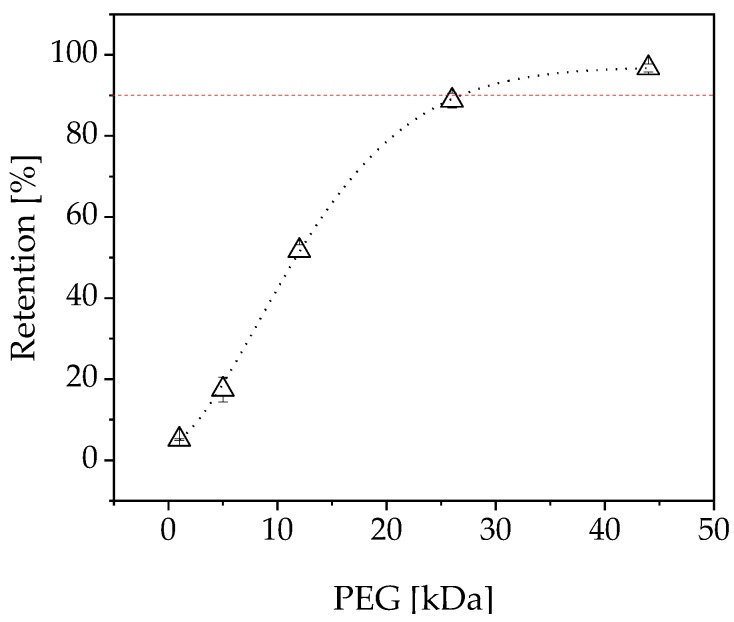
Sieve curve of the used membrane (atech innovations). PEG = polyethylene glycol.

To determine the MWCO of the membrane, a sieve curve was recorded from the measured PEG peak areas of the permeate and retentate (Figure 11). The MWCO was determined by regression analysis. This revealed a MWCO of 26.78 kDa, which is compatible with EMRs containing 1-FFT because the molecular mass of the enzyme is 87.5 kDa.

### 4.10. Continuous FOS Production

**Figure 12:**
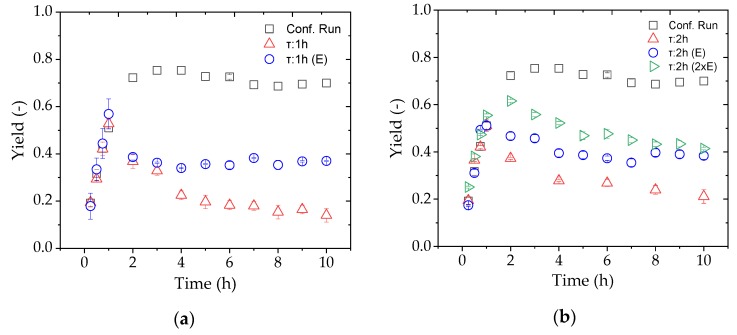
The yield of FOS during continuous production in an enzyme membrane reactor (EMR) after switching from total-recycle to continuous mode after 1 h with residence times of (**a**) 1 and (**b**) 2 h. Reactions were tested without enzyme replacement (red), with a single dose of replacement enzyme (blue), and with a double dose of replacement enzyme (green).

Finally, we evaluated a continuous process for the production of FOS by adding the substrate solution and changing the TMP to achieve a constant flux with reference to a residence time of 1 or 2 h. In order to compensate for the enzyme lost during the reaction, a second pump was used to add the appropriate amount of fresh enzyme solution based on the known concentration of the enzyme stock. Figure 12 shows the evaluation of the continuous processes at different residence times based on the FOS yield, comparing reactions with and without the addition of further enzyme.

Compared to the confirmation run in total-recycle mode, none of the set residence times with and without additional enzyme achieved the desired yield. In the first hour, the yields were similar because the process conditions were the same, but the enzyme was rapidly washed out immediately after changing the process mode, especially with a residence time of 1 h. The yield was higher compared to the residence time of 2 h because less of the enzyme was lost through the membrane due to the lower flux. The addition of enzyme solution achieved the partial but not full recovery of enzyme activity compared to total-recycle mode. Given that the longer residence time and addition of enzymes still did not achieve a sufficient yield, the amount of additional enzyme was doubled. Although the yield increased during the first hours of the continuous process, it had decreased again by 8 h and was only slightly higher than the single-dose treatment after 10 h. 

## 5. Discussion

Our preliminary experiments in an EMR containing the enzyme 1-FFT revealed an unavoidable decline in flux, so we applied a D-optimal design using the permeate flux after 8 h as a parameter and statistically optimized the model with respect to the enzyme activity. A novel EMR system based on ceramic membranes has been described for the production of galacto-oligosaccharides, and the authors reported a drop in flux from 23 to 11 L m^−2^ h^−1^ after 3 h at a TMP of 2 bar [38]. Similarly, the flux in our experiments fell to 11.87 ± 1.89 L m^−2^ h^−1^ after filtration for 8 h in the confirmation run. The contour plot of the D-optimal design for the permeate flux (Figure 3a) reveals a local maximum at low TMP and a moderate CFV. With respect to concentration polarization, a higher TMP would increase the filtration resistance and thus reduce the permeate flux. A further increase in the TMP can lead to fouling [39], additionally in our experiments we clearly observed that a further increase in the TMP causes a loss of enzyme activity (Figure 3b).

We investigated the loss of flux in more detail, initially by testing different cleaning strategies. We found that sequential cleaning with P3 Ultrasil 14 and citric acid was necessary to remove all reversible fouling, but that the effect of citric acid was more important. These results suggest that P3 Ultrasil 14 removes an organic gel layer and allows citric acid better access to unblock the membrane pores.

The high temperature of the filtration (70 °C) caused the solution to turn brown, so we measured the absorbance of the solution to determine the influence of the Maillard reaction. We observed a linear increase in absorbance throughout the reaction, which therefore could not explain the rapid loss of flux within the first hour of the filtration run following the addition of the enzyme solution. Most substrate conversion takes place during the first 2 h, which suggests that the observed phenomenon may be linked to the formation of FOS. However, we observed no change in the FOS concentration over 8 h during the investigation without the presence of enzyme in the filtration solution (Figure 7). The flux measured during this experiment was 22.67 L m^−2^ h^−1^ (191% higher than the confirmation run), which implied that previously extracted proteins or an interaction between the extracted protein and the FOS product may be responsible for the fouling in EMR mode. The flux measured in the enzyme stability experiment was 248.6 L m^−2^ h^−1^ with 10% (*v*/*v*) enzyme solution in the absence of sugar, which implies no enhanced resistance due to the used enzyme.

Recent work to investigate fouling phenomena in membrane bioreactor systems has shown that the major fouling mechanism is cake layer resistance, especially when the feed is a whole-cell suspension [25,40,41]. Various terms are used in the literature to describe cake layer formation, including gel layer formation by bacterial cells [42,43], colloids [44,45], and enzymes [46]. Using the resistance-in-series model, we found that a gel layer was present but was only responsible for 3.7% of the total resistance. The adsorption of salts may also add to the resistance, which may explain the necessity to clean the membrane with citric acid. The resistance-in-series model showed that the largest component (40.2%) was attributable to unknown resistance or a complex combination of fouling mechanisms.

Flux modeling showed that our data fitted the dead-end filtration equations. The low overflow velocity of the experiments resulted in a low Reynolds number, especially in the confirmation run (Re = 287.33). The best fit was observed for the complete blocking model, which implies a blockage in the pores. We therefore carried out sieve analysis, which clearly showed a small pore size of 26.78 kDa compared to the enzyme size of 85 kDa. The resistance to filtration resulting from colloids with a smaller average size than the MWCO of the membrane can lead to partial or total pore clogging according to the complete blocking and standard blocking flux models [25,47]. This was confirmed by our results, given that the resistance-in-series and enzyme stability experiments indicated that the enzymes are not solely responsible for the loss of flux during filtration. Even so, a combination of initial pore blocking by the FOS product plus additional fouling by the enzyme appears to be the most appropriate explanation for our results. We propose that the enzymes and FOS block the pores, and that this process cannot be avoided within the D-optimal design space. Our proposed fouling mechanism involving interactions between proteins and polysaccharides is supported by previous research showing interactions among other mixtures of foulants [48].

The yields achieved in the confirmation run of the D-optimal design could not be repeated by continuous enzyme catalysis (Figure 12). The variation of the residence time between 1 and 2 h showed no influence on the yield. With an additional enzyme feed, the yield was doubled at both residence times. The addition of a double dose of enzyme (τ = 2 h) did not increase the yield after 10 h in the steady state. From these results we conclude that the enzyme deactivation rate was higher than the feed rate.

## 6. Conclusions

We developed a successful EMR system for continuous FOS production with two alternative residence times. These were established by improving the flux using a D-optimal design that also reduced the loss of enzyme activity. A statistically optimized process point at a TMP of 0.5 bar and a CFV of 0.6 m s^−1^ could be identified within the design space. Further research is required to realize industrial-scale FOS production, specifically to improve the FOS yield and the separation of by-products and unreacted substrate. Several fouling phenomena responsible for the decline in flux were investigated, revealing that complex fouling mechanisms, probably involving an interactions of proteins and polysaccharides, cause complete pore blocking. Although we were able to exclude some fouling phenomena, more work is required to determine the specific fouling mechanisms that reduce the flux in our EMR system. 

## Figures and Tables

**Table 1:** Mathematical formulation of fouling phenomena for dead-end filtration, based on the published definitions of J_0_, n, and k [27].

Fouling Mechanism	n	Fouling Equation	Equation Number
complete pore blocking	2	$J\;=\;J_{0}\times \exp\left({-k\;\times \;J}_{0}\times \;t\right)$	(4)
standard pore blocking	1.5	$J\;=\;\frac{J_{0}}{{\left({1\;+\;J}_{0}\times \;k\;\times \;t\right)}^{2}}$	(5)
intermediate pore blocking	1	$J\;=\;\frac{J_{0}}{{1\;+\;k\;\times \;J}_{0}\times \;t}$	(6)
cake filtration	0	$J\;=\;\frac{J_{0}}{{\left({1\;+\;k\;\times \;J}_{0}\times \;t\right)}^{0.5}}$	(7)

**Table 2:** Mathematical formulation of fouling phenomena for cross-flow filtration, based on the published definitions of J_0_, J_ss_, n, and k [29].

Fouling Mechanism	n	Fouling Equation	Equation Number
complete pore blocking	2	${J\;=\;J}_{\mathrm{ss}}+\;\left(J_{0}{-\;J}_{\mathrm{ss}}\right)\;\exp\left(-k\;t\right)$	(9)
standard pore blocking	1.5	$\frac{1}{J^{0.5}}\;=\;\frac{1}{J_{0}^{\;0.5}}\;+\;k\;t$	(10)
intermediate pore blocking	1	$k\;t\;=\;\frac{1}{J_{\mathrm{ss}}}\ln\left(\frac{J}{J_{0}}\frac{{(J}_{0}\;{-\;J}_{\mathrm{ss}})}{\left({J\;J}_{\mathrm{ss}}\right)}\right)$	(11)
cake filtration	0	$k\;t\;=\;\frac{1}{J_{\mathrm{ss}}^{2}}\left[\ln\left(\frac{J_{0}}{J}\;\frac{{(J}_{0}\;{-\;J}_{\mathrm{ss}})}{\left({J\;-\;J}_{\mathrm{ss}}\right)}\right)\right]{-\;J}_{\mathrm{ss}}\left(\frac{1}{J}\;-\;\frac{1}{J_{0}}\right)$	(12)

**Table 3:** Properties of the ceramic membrane used in this study.

Property	Value
manufacturer	atech innovations
type	37/2
support layer	Al_2_O_3_
active layer	TiO_2_
pH stability	0–14
cut-off	20 kDa
length	970 mm
number of channels	37
inner diameter of each channel	2 mm
outer diameter of each channel	25.4 mm
filtration area	0.226 m^2^
cross-flow area	1.1624 × 10^−4^ m^2^

**Table 4:** Cleaning strategies.

Cleaning Strategies	Cleaning Sequence
A	C 2 h
B	U 2 h
C	C 2 h → U 2 h
D	U 2 h → C 2 h
E	C 2 h → U 2 h → C 2 h

Each step was conducted for 2 h. C = citric acid, U = P3 Ultrasil 14.

**Table 5:** Calculated resistance values.

Membrane Resistance	Resistance (1 × 10^12^ m^−1^)	Filtration Solution	Viscosity at 70 °C (mPa)	Flux_t = 8h_ (L m^−^² h^−1^)
R_total_	3.01	Catalysis solution	5.05	13.6
R_m_	1.68	Water	0.40	265.0
R_irrev_	0.21	Water	0.40	308.0
R_a+cp_	0	Sucrose (600 g L^−1^) + FM 10% (*v*/*v*)	5.05	53.5
R_g_	0.11	Enzyme solution 10 % (*v*/*v*)	0.40	248.6
R_unknown_	1.22	Calculated	-	-

FM = Fresh fermentation medium (uncultivated); R_total_ = total resistance; R_m_ = membrane resistance; R_irrev_ = irreversible resistance still present after cleaning; R_a+cp_ = resistance caused by adsorption and concentration polarization of the substrate solution; R_g_ = resistance of the gel layer caused by filtration of the enzyme solution; R_unknown_ = unknown resistance.

**Table 6:** R^2^ values for both dead-end and crossflow filtration models.

Filtration Type	TMP (bar)	CFV (m s^−1^)	Complete Blocking, R^2^	Standard Blocking, R^2^	Intermediate Blocking, R^2^	Cake Filtration, R^2^
Dead-end	0.5	0.60	0.966	0.931	0.874	0.745
	2.75	0.68	0.937	0.890	0.831	0.691
Cross-flow	0.5	0.60	0.952	0.874	0.842	0.663
	2.75	0.68	0.935	0.831	0.827	0.642

## Abbreviation

FOS	Fructo-oligosaccharides
1-FFT	Fructosyltransferase
EMR	Enzyme membrane reactor
GF_2_	1-kestose
GF_3_	Nystose
GF_4_	1F-fructofranosylnystose
UF	Ultrafiltration
TMP	Transmembrane pressure (bar)
DoE	Design of experiments
CFV	Cross-flow velocity (m s^−^^1^)
R_total_	Total resistance (m^−^^1^)
J	Permeate flux (L m^−2^ h^−1^)
η	Permeate dynamic viscosity (Pa s)
ΔP	Pressure difference during filtration (Pa)
R_m_	Membrane resistance (m^−1^)
R_a_	Adsorption resistance (m^−1^)
R_cp_	Concentration polarization resistance (m^−1^)
R_g_	Gel layer resistance (m^−1^)
*n*	model constant (-)
*k*	Fouling-dependent coefficient (-)
t	Time (s)
J_0_	Initial flux (L m^−2^ h^−1^)
J_ss_	Steady-state flux (L m^−2^ h^−1^)
C	Citric acid
U	P3 Ultrasil 14
BCA	Bicinchoninic acid
MWCO	Molecular weight cut-off
PEG	Polyethylene glycol
